# Species-level microbiota of ticks and fleas from *Marmota himalayana* in the Qinghai-Tibet Plateau

**DOI:** 10.3389/fmicb.2023.1188155

**Published:** 2023-06-21

**Authors:** Lingzhi Dong, Yaben Li, Caixin Yang, Jian Gong, Wentao Zhu, Yuyuan Huang, Mimi Kong, Lijun Zhao, Feifei Wang, Shan Lu, Ji Pu, Jing Yang

**Affiliations:** ^1^Department of Epidemiology, School of Public Health, Shanxi Medical University, Taiyuan, China; ^2^State Key Laboratory of Infectious Disease Prevention and Control, Chinese Center for Disease Control and Prevention, National Institute for Communicable Disease Control and Prevention, Beijing, China; ^3^Department of Infectious Diseases and Clinical Microbiology, Beijing Chao-Yang Hospital, Capital Medical University, Beijing, China; ^4^Research Units of Discovery of Unknown Bacteria and Function, Chinese Academy of Medical Sciences, Beijing, China

**Keywords:** tick, flea, *Marmota himalayana*, *Anaplasma phagocytophilum*, *Wolbachia*, *Bartonella*

## Abstract

**Introduction:**

Ticks and fleas, as blood-sucking arthropods, carry and transmit various zoonotic diseases. In the natural plague foci of China, monitoring of *Yersinia pestis* has been continuously conducted in *Marmota himalayana* and other host animals, whereas other pathogens carried by vectors are rarely concerned in the Qinghai-Tibet Plateau.

**Methods:**

In this study, we investigated the microbiota of ticks and fleas sampling from *M. himalayana* in the *Qinghai-Tibet* Plateau, China by metataxonomics combined with metagenomic methods.

**Results:**

By metataxonomic approach based on full-length 16S rDNA amplicon sequencing and operational phylogenetic unit (OPU) analyses, we described the microbiota community of ticks and fleas at the species level, annotated 1,250 OPUs in ticks, including 556 known species and 492 potentially new species, accounting for 48.50% and 41.71% of the total reads in ticks, respectively. A total of 689 OPUs were detected in fleas, consisting of 277 known species (40.62% of the total reads in fleas) and 294 potentially new species (56.88%). At the dominant species categories, we detected the *Anaplasma phagocytophilum* (OPU 421) and potentially pathogenic new species of *Wolbachia, Ehrlichia, Rickettsia*, and *Bartonella*. Using shotgun sequencing, we obtained 10 metagenomic assembled genomes (MAGs) from vector samples, including a known species (*Providencia heimbachae* DFT2), and six new species affliated to four known genera, i.e., *Wolbachia, Mumia, Bartonella*, and *Anaplasma*. By the phylogenetic analyses based on full-length 16S rRNA genes and core genes, we identified that ticks harbored pathogenic *A. phagocytophilum*. Moreover, these potentially pathogenic novel species were more closely related to *Ehrlichia muris, Ehrlichia muris* subsp. *eauclairensis, Bartonella rochalimae*, and *Rickettsia limoniae*, respectively. The OPU 422 Ehrlichia sp1 was most related to *Ehrlichia muris* and *Ehrlichia muris subsp. eauclairensis*. The OPU 230 *Bartonella* sp1 and *Bartonella* spp. (DTF8 and DTF9) was clustered with *Bartonella rochalimae*. The OPU 427 *Rickettsia* sp1 was clustered with *Rickettsia limoniae*.

**Discussion:**

The findings of the study have advanced our understanding of the potential pathogen groups of vectors in marmot (*Marmota himalayana*) in the Qinghai-Tibet Plateau.

## Introduction

Vector-borne diseases (VBDs) are infections that are primarily transmitted through an invertebrate, generally, insects. With the global climatic alteration and human activity amplification, the VBDs pose a significant burden on public health and economic development. It is estimated that 80% of the population in the world is at risk of two or more VBDs. They account for 17% of all infectious diseases and cause more than 700,000 deaths annually (Golding et al., 2015; WHO, 2017; Chala and Hamde, 2021). As the largest developing country in the world, China has made great progress in the prevention and treatment of infectious diseases. However, emerging infectious diseases are a new challenge for China (Wang et al., 2008).

Ticks are obligate blood-sucking arthropods that can parasitize vertebrates and transmit a wide variety of infectious microorganisms such as viruses, bacteria, spirochetes, protozoans, and parasites (Parola, 2004). As a major contributor to vector-borne diseases, a huge number of novel tick-borne disease agents have been reported such as borreliosis, ehrlichioses, anaplasmosis, and tick-borne rickettsial diseases. Among the 109 tick-borne pathogens identified in China (Zhao et al., 2021) between 1950 and 2018, 65 were newly identified. Plenty of human cases have been confirmed for tick-borne infections with *Borrelia, Anaplasmataceae, Babesia*, and spotted fever group *Rickettsiae* (Fang et al., 2015). Fleas are blood-sucking arthropods that parasitize mammals and birds. They are known for transmitting flea-borne *Yersinia pestis* causing plague (Zeppelini et al., 2016). In recent years, increasing numbers of flea-borne diseases, including murine typhus, epidemic typhus, and flea-borne spotted fever have been reported across the world (Bitam et al., 2010). Fleas have also been reported to transmit *Bartonella*, including *Bartonella henselae*, the agent cat-scratch disease (Chomel et al., 2006; Gutiérrez et al., 2015).

Marmots (*Marmota himalayana*) were identified as the predominant host of *Yersinia pestis* in the Qinghai-Tibet Plateau, China (Ben-Ari et al., 2012). In the natural plague foci of China, such as Qinghai and Xinjiang, marmots and other animal hosts have been continuously monitored under China's Plague Surveillance Program (Qin et al., 2022). Our previous studies have found the possibility of marmots to be hosts and reservoirs for pathogens including tick-born encephalitis virus (Dai et al., 2018) and delta-Coronaviruses (Zhu et al., 2021). In recent years, there have been some studies on vector-borne pathogens in this area, such as the research of spotted fever group *Rickettsia* infecting ticks, yaks, and Tibetan sheep (He et al., 2021b) and the molecular detection of *Anaplasm, Babesia*, and *Theileria* in yaks and Tibetan sheep (He et al., 2021a). However, the characterization of species-level microbiota of vectors, especially in fleas, is lacking in marmots. In the present study, we described the microbiota community of ticks and fleas sampling from marmots in Qinghai-Tibet Plateau, China. We aimed to discover the profile of tick-borne and flea-borne pathogens or potentially new pathogenic bacterial species, providing etiological information for vector infectious disease prevention and control.

## Materials and methods

### Samples collection

The ticks and fleas were collected from *M. himalayana* in Tongren, Huangnan Tibetan Autonomous Prefecture (Altitude: 3273 m; N: 35°49′, E: 102°28′), Qinghai Province, China, from July to September 2018. Marmots were collected for routine surveillance and screened for plague in Autumn 2018. All the marmots were captured and preliminarily detected *Y. pestis* F1 antibody by the colloidal gold method. Then, ticks and fleas parasitized on the surface of marmots were collected and stored on the principle of one marmot per frozen tube. All the vectors were transported to the local lab, and subsequently, ticks and fleas were identified as adults by professionals using a DM-500 binocular biological microscope, referring to the reference book of Cai et al. (1997). All the samples were transported to our laboratory in Beijing and stored at −80°C prior to DNA extraction. The sampling work was conducted by Qinghai Institute for Endemic Disease Prevention and Control for seasonal vector surveillance. The ethical practice was approved by the Ethical Committee of the National Institute for Communicable Disease Control and Prevention, Chinese Center for Disease Control and Prevention, China (No. ICDC-2018004).

### DNA extraction and full-length 16S rDNA amplification sequencing

A total of 25 ticks and 24 fleas were enrolled in our experiment, which were divided into eight pools for each sample ticks and fleas according to the engorgement level (Supplementary Table S1). Ticks and fleas were surface-sterilized in 10% bleach and then washed twice in 99% ethanol to remove bacteria from the external body. Sixteen vector samples and two blank control groups were homogenized (Liu et al., 2017; Adegoke et al., 2020) separately in the PBS solution by the TissueLyser II (Qiagen, Germany) at 24 Hz, 37°C for 2 min, three times. The TissueLyser II system was achieved through high-speed shaking with beads, which beat and grid samples, and also simultaneously homogenized samples to facilitate subsequent purification procedures. The total DNA was then extracted from the supernatant using the QIAamp DNA Mini Kit (Qiagen, Germany). The quality of DNA extraction was checked by NanoDrop 2000 (Thermo Fisher Scientific, USA). The almost full-length 16S rRNA gene was amplified using the universal primers 27F/1492R with 16 nt symmetric (reverse complement) barcodes tagged at the 5′ end, as described previously (Yang et al., 2020). The libraries of PCR products were generated, followed by sequencing on the PacBio Sequel platform at Tianjin Biochip Corporation, China.

The raw sequences generated were filtered and cleaned using the previous pipeline described by Meng et al. (2017) and Yang et al. (2020), i.e., the raw sequencing data were initially processed based on the PacBio SMRT Link (version 6.0.0) pipeline, QIIME (Bolyen et al., 2019) was used to split data and filter out adapters, primers, ambiguous bases, and chimeras were detected and removed using the UCHIME (Edgar et al., 2011) algorithm implemented in USEARCH (version 11.0.667; option: -uchime_ref -strand plus -nonchimeras) with the RDP Gold reference database.

### Species-level taxonomy by operational phylogenetic unit analyses

High-quality sequences of the full-length 16S rRNA gene were clustered into operational taxonomic units (OTUs) with an identity threshold of 98.7% by the USEARCH pipeline. Then, all the representative sequences for each OTU were grouped into operational phylogenetic units (OPUs) using the tool Arb by the visual inspection of the final phylogenetic trees. The reference database used was LTP132 (which was the latest version at the time of this study). The detailed analysis process and classification criteria of OPU were shown in our previous studies (Meng et al., 2017; Bai et al., 2018; Yang et al., 2020). In brief, each OPU consisting of one or more OTUs can be equated to a species-level taxon. When an identity value of OTU representatives with a type strain was >98.7%, it was identified as known species. A putative new species within a specific genus was defined when the identity values of OTU representatives with the closest type strain were <98.7 and >94.5%. When OTUs represent a sequence whose membership position was not clear but was closely affiliated to a known genus, family, or higher taxa, it was listed as a potential new “higher taxa.” Taxa around the OTU sequences that were represented by a large number of uncultured organisms and were not attached to any of the internal taxa were referred to as “uncultured taxa” categories.

### Shotgun metagenomic sequencing, assembly, and contig binning

The DNA material used for shotgun metagenomic sequencing was the same as that described in 16S rRNA-based amplicon sequencing section. DNA degradation, contamination, and concentration were monitored by agarose gel electrophoresis and Qubit^®^ 2.0 Fluorometer. Then, DNA libraries were constructed, purified, analyzed, quantified, and sequenced on the Illumina PE150 platform (150-bp, paired-end) at the Novogene Co., Ltd. (Beijing, China).

The raw paired-end reads were quality-filtered to remove the adapter and low-quality sequences using the Readfq (V8, https://github.com/cjfields/readfq). Then, the reads mapped to the host genomic DNA by Bowtie2 (version 2.2.4, http://bowtie-bio.sourceforge.net/bowtie2/index.shtml) with the following parameter settings: –end-to-end, –sensitive, -I 200, and -X 400 were filtered out. The sequence data were assembled individually by MEGAHIT (version 1.0.4, https://github.com/voutcn/megahit) using the parameter settings: –presets meta-large (–end-to-end, –sensitive, -I 200, -X 400). Next, the assembled contigs were binned with three different tools using the default settings in MetaWRAP (Uritskiy et al., 2018) (version 1.3.2) pipeline, and the completeness and contamination of the reassembled metagenomic assembled genomes (MAGs) were estimated by CheckM (Parks et al., 2015) (version 1.0.13). The reassembled MAGs were classified using the Genome Taxonomy Database Toolkit (GTDB-Tk version 1.3.0) based on the GTDB RefSeq_95 database. The taxonomic assignment was defined by a combination of the placement of the query genome in the GTDB reference tree, the RED of the query genome, and Average Nucleotide Identity (ANI) (Ciufo et al., 2018; Jain et al., 2018) to reference genomes.

### Phylogenetic analyses of the potential vector-borne pathogens

The phylogenetic analyses of full-length 16S rRNA gene amplicon sequencing were performed with MEGA X (https://www.megasoftware.net/) using the maximum-likelihood (ML) algorithm. Multiple alignments were performed with the program CLUSTAL_W, and all cases were evaluated based on 1,000 bootstrap replicates. The phylogenetic analyses of reassembled MAGs were performed with FastTree (Price et al., 2009) by using the neighbor-joining (NJ) algorithm, which was generated based on core genes that were extracted from the reference whole genomes and visualized in Dendroscope 3.0.

## Results

### Species-level microbiota of ticks and fleas

A total of 16 vector pools were sequenced, with eight pools each for ticks and fleas (Supplementary Table S1). The PacBio sequel sequencing platform rendered a total of 135,040 and 98,090 raw reads for ticks and fleas, respectively. After quality filtering and chimera removal, a total of 110,760 (80.50%) and 70,502 (71.10%) high-quality reads were obtained from ticks and fleas, with an average of 13,845.00 ± 6,588.00 per tick pool and 8,812.75 ± 2,681.68 per flea pool. The average length was 1,437.00 ± 15.07 and 1,453.63 ± 7.02 nucletide (nt) per read for ticks and fleas, respectively (Supplementary Table S2). The near full-length 16S rRNA gene sequences were clustered into 4,789 and 2,170 OTUs in respective ticks and fleas at an identity threshold of 98.7% using the USEARCH pipeline. Subsequently, a total of 1,250 and 689 operational phylogenetic units (OPUs) were obtained for ticks and fleas, respectively, with an average of 303.25 ± 164.84 per tick pool and 153.36 ± 96.37 per flea pool (Supplementary Table S3). We further classified these OPUs into four categories: (i) known species (556 OPUs, relative abundance 48.50% vs. 277 OPUs, 40.62% in ticks and fleas, respectively); (ii) potentially new species (492 OPUs, 41.71% vs. 294 OPUs, 56.88%); (iii) higher taxa (133 OPUs, 6.19% vs. 83 OPUs, 2.02%); and (iv) uncultured bacteria (69 OPUs, 3.60% vs. 35 OPUs, 0.48%; Supplementary Table S3 and Supplementary Figure S1). It can be noted that the vast majority of the flora was identified at the species level (known species and potentially new species), indicating the relative abundance of dominant taxa reached 90.21% and 97.50% in ticks and fleas, respectively.

### The potential vector-borne pathogens detected in ticks and fleas at the species level

**Figure 1:**
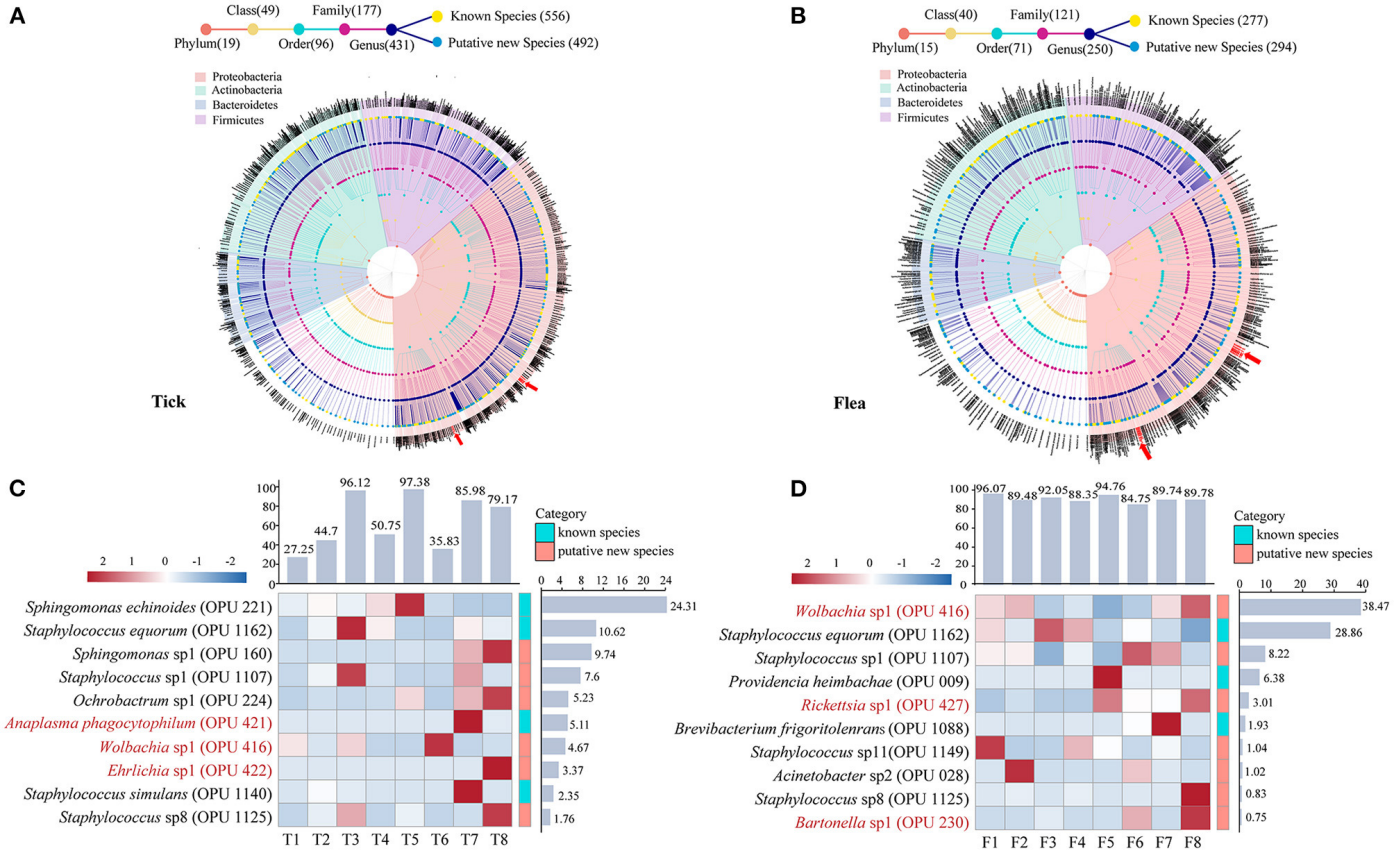
Taxonomic profile and dominant taxa in ticks and fleas at species level. Taxonomic trees of **(A)** 556 known species and 492 putative new species in ticks and **(B)** 277 known species and 294 putative new species in fleas. The heat map and relative abundance of the top ten abundant bacteria at the species level **(C)** in ticks and **(D)** in fleas. Each dot represents a taxonomic entity. From the inner to the outer circles, the taxonomic levels range from phylum to species. Dots of different colors indicate different taxonomic levels according to the color key shown. The total number at different hierarchical levels is displayed in brackets. The potential pathogens are indicated by red arrows and words in the figure. Different colored squares represent different phyla according to the color key shown.

In ticks, the 556 known species and 492 putative new species affiliated to 19 phyla, 49 classes, 96 orders, 177 families, and 431 genera, accounting for 90.21% of the total reads (Figure 1A and Supplementary Figure S1). The top 10 most abundant species in ticks represented 74.76% of the total reads, ranging from 27.25% to 97.38% in individual tick pools (Figure 1C). Of these 10 species, the *Anaplasma phagocytophilum* (OPU 421) and *Ehrlichia* sp1 (OPU 422) were presented, and their relative abundance were 5.11% and 3.37%, respectively, and which were indicated by red arrows and words in Figures 1A, C. However, *Bartonella* sp1 (OPU 230) had a relatively lower abundance of 0.01%. In flea samples, most of the reads (97.50%) were annotated to the species level, including 277 known species and 294 putative new species (Figure 1B and Supplementary Figure S1). The top 10 species accounted for 90.51% of the total reads, ranging from 84.51% to 96.07% in individual flea pools (Figure 1D). The potential novel species affiliated to vector-borne pathogenic genera including *Rickettsia* and *Bartonella* were founded in these top 10 species, and their relative abundance were 3.01% and 0.75%, respectively.

In addition, *Wolbachia* were detected in the top 10 abundance at the species level in both ticks and fleas. The relative abundance of *Wolbachia* sp1 (OPU 416) were 4.67% in ticks and 38.47% in fleas.

**Figure 2:**
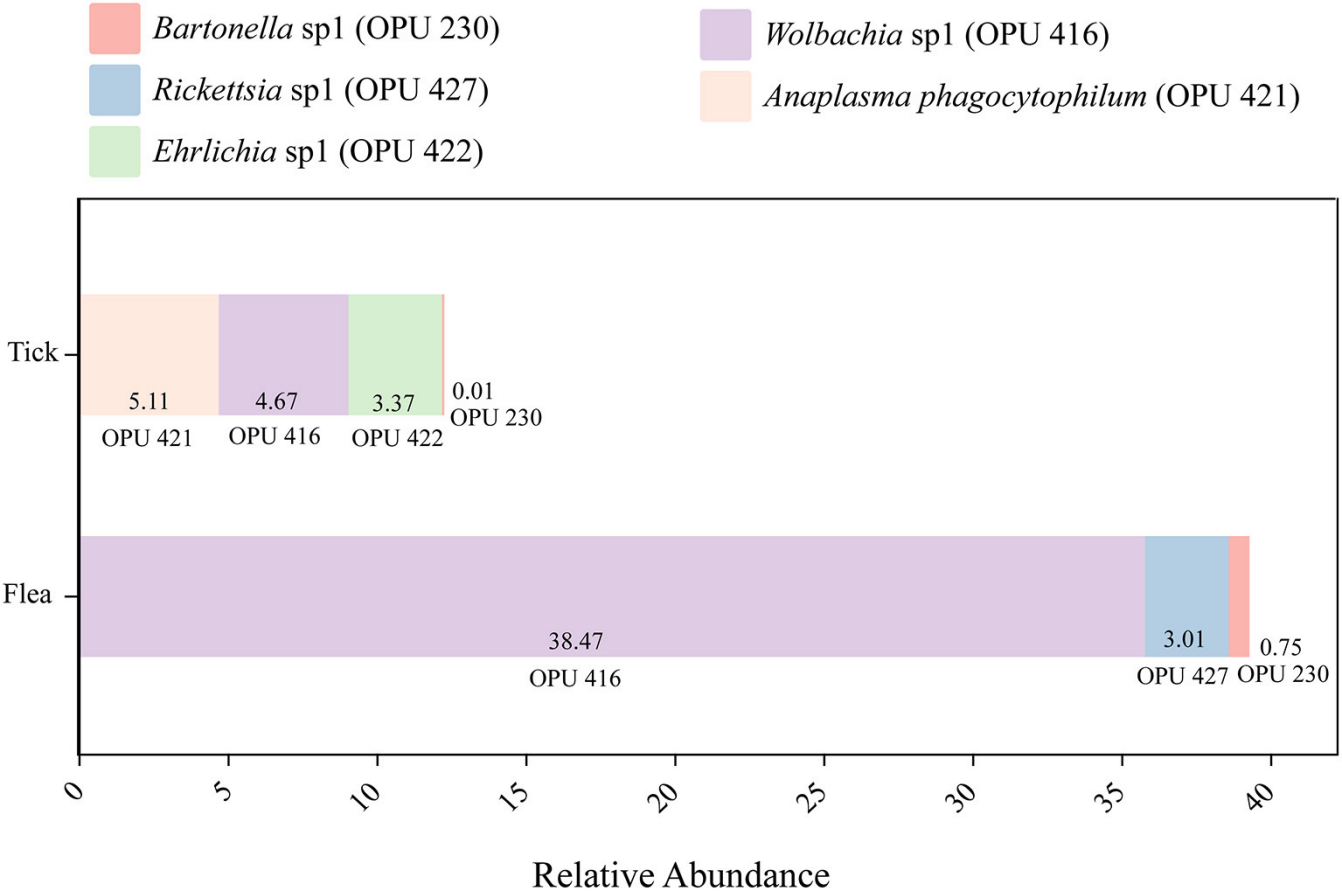
The relative abundance of the potential pathogens in ticks and fleas including *Anaplasma phagocytophilum* (OPU 421), *Ehrlichia* sp1 (OPU 422), *Bartonella* sp1 (OPU 230), *Rickettsia* sp1 (OPU 427), and *Wolbachia* sp1 (OPU 416).

Notably, the known vector-borne pathogen *A. phagocytophilum* and potentially novel species affiliated with vector-borne pathogenic genera, including *Ehrlichia, Rickettsia*, and *Bartonella* were detected in the dominant species categories of the microbiota community in ticks and fleas, accounting for 13.16% and 42.23% of the total reads in ticks and fleas, respectively (Figure 2). Hence, we intend to acquire the complete genomes of these potential pathogens for deep analysis.

### The metagenomic assembled genomes of ticks and fleas

We used the same DNA samples as full-length 16S rDNA sequencing for shotgun metagenomic sequencing. An average of 7,040.24 ± 747.37 and 7,396.88 ± 627.33 Mb high-quality reads data (paired-end, 150-bp) per sample pools were generated in ticks and fleas, respectively. The *de novo* assembly produced a mean of 474,197 ± 48,301 and 287,426 ± 84,212 kb contigs per sample pool comprising respective ticks and fleas (Supplementary Table S4). Finally, we recovered nine MAGs (bins) from the assembled contigs in fleas and one MAG in ticks. Detailed descriptions of the 10 MAGs and their proposed nomenclatures were provided in Supplementary Table S5. A total of six out of 10 MAGs had >90% completeness and <5% contamination, while three MAGs showed >70% completeness and <10% contamination, and one MAG had <50% completeness and <15% contamination (Supplementary Table S5). Moreover, 10 MAGs (>1 Mbp) were subsequently identified using the Genome Taxonomy Database Toolkit (GTDB-Tk) combined with ANI value calculation. It was shown that one MAG (f5_bin2) belonged to known bacterial species (*Providencia heimbachae* DFT2; >95% ANI with a complete genome), six MAGs could be assigned to undescribed species affiliated to four known genera, including *Wolbachia, Mumia, Bartonella*, and *Anaplasma*, and the remaining three MAGs had low ANI values (<85%), demonstrating that they may belong to the family *Rickettsiaceae* (f5_bin1, f6_bin6) and order *Acidimicrobiales* (f6_bin5).

### Phylogenetic analyses of the potential vector-borne pathogens

**Figure 3:**
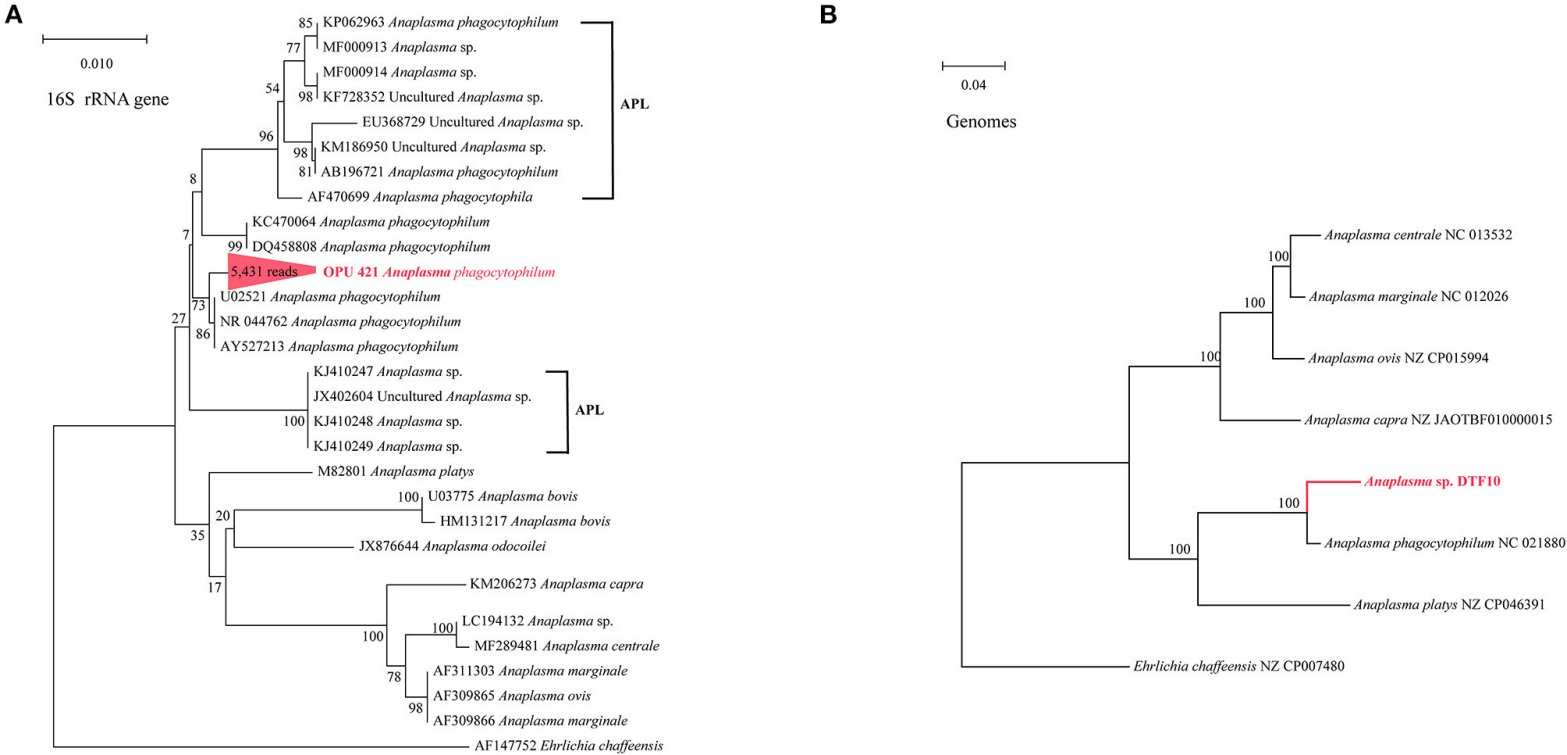
Phylogenetic trees based on full-length 16S rRNA gene sequences and genomic core genes revealed the positions of **(A)** the OPU 421 *Anaplasma phagocytophilum* **(B)** the *Anaplasma* sp. DTF10 (208 core genes). The phylogenetic trees were constructed using maximum-likelihood algorithms based on full-length 16S rRNA gene sequences and neighbor-joining (NJ) algorithms based on core genes. The APL represents genetically related *A. phagocytophilum*-like (APL) species. Trees showed the precise position of the OPUs and genomes of the identified species compared with members of the most closely related reference sequences. Red labels are the OPUs and genomes of the identified species, while known species are shown in black.

We identified the known vector-borne pathogen *A. phagocytophilum* and potentially new species affiliated with vector-borne pathogenic genera, including *Ehrlichia, Rickettsia*, and *Bartonella*, using full-length 16S rRNA gene sequencing. Further through metagenomic data, we recovered the MAGs of *Bartonella* and *Anaplasma* from fleas and ticks. To further confirm the taxonomic positions of these potential vector-borne pathogens, we constructed the phylogenetic trees based on the full-length 16S rRNA genes and core genes using the ML and NJ algorithms, respectively. The OPU 421 identified in ticks with 5,431 reads affiliated as an independent cluster with *A. phagocytophilum* (U02521, NR 044762, and AY537213) with an average 16S rRNA gene sequence similarity of 99.72%, possibly representing a known species *A*. *phagocytophilum* (Figure 3A). The phylogenomic tree based on 208 core genes showed that *Anaplasma* sp. DTF10 (tick_bin1) formed a branch with *A. phagocytophilum* (NC 021880; Figure 3B). The ANI value between *Anaplasma* sp. DTF10 and *A. phagocytophilum* NC 021880 was 89.3%, indicating that it could be assigned as a new species of *Anaplasma*. Phylogenetic analysis based on the 16S rRNA gene sequences revealed that OPU 422 (3,586 reads in ticks) was related to *Ehrlichia muris* (HM543745, NRO25962, NR121714, and U15527) and *Ehrlichia muris* subsp. *eauclairensis* (HM543745; **Figure 5A**) with a maximum 16S rRNA gene sequence similarity of 98.4%. Thus, OPU 422 could represent a new species belonging to the genus *Ehrlichia*.

**Figure 4:**
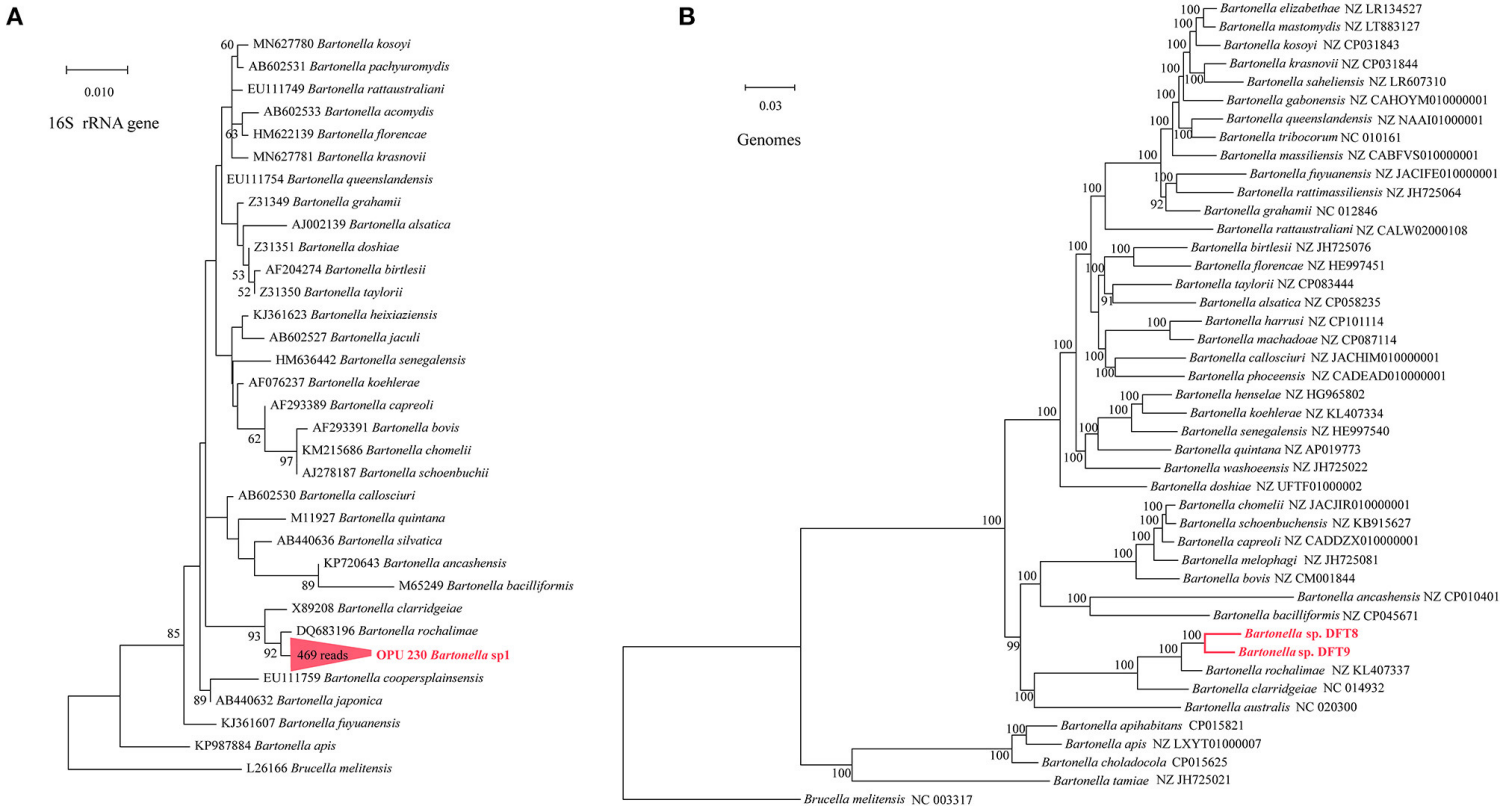
Phylogenetic trees based on full-length 16S rRNA gene sequences and genomic core genes revealed the positions of **(A**) the OPU 230 *Bartonella* sp1 and **(B)** the *Bartonella* sp. DTF8 and the *Bartonella* sp. DTF9 (333 core genes). The phylogenetic trees were constructed using the maximum-likelihood and neighbor-joining (NJ) algorithms based on full-length 16S rRNA gene sequences and core genes. Trees showed the precise position of the OPUs and genomes of the identified species compared with members of the most closely related reference sequences. Red labels are the OPUs and genomes of the identified species, while known species are shown in black.

**Figure 5:**
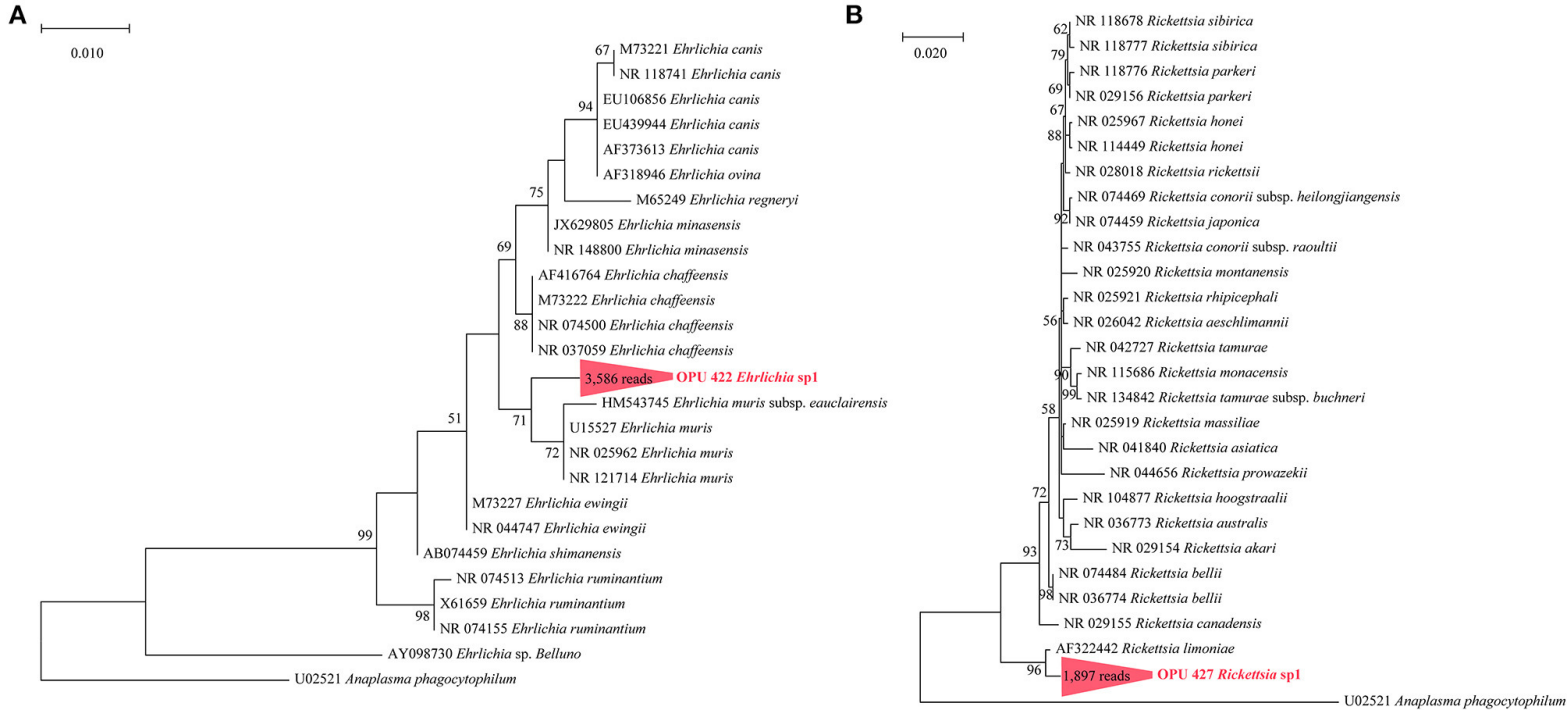
Phylogenetic trees based on full-length 16S rRNA gene sequences revealed the positions of **(A)** the OPU 422 *Ehrlichia* sp1 and **(B)** the OPU 427 *Rickettsia* sp1. The phylogenetic trees was constructed using a maximum-likelihood algorithm based on full-length 16S rRNA gene sequences. Trees showed the precise position of the OPUs of the identified species compared with members of the most closely related reference sequences. Red labels are the OPUs of the identified species, while known species are shown in black.

The OPU 230 (469 reads in fleas) were placed in a clade with *Bartonella rochalimae* (DQ683196) based on the phylogenetic analyses of the 16S rRNA gene (Figure 4A). The representative sequences of OPU 230 *Bartonella* sp1 showed an average identity of 98.40 ± 0.01% with *B. rochalimae* (DQ683196). A phylogenomic tree based on 333 core genes showed that the two MAGs (f6_bin8, f8_bin9) of *Bartonella* spp. (DTF8 and DTF9) formed a distinct clade and were clustered with *B*. *rochalimae* (NZ KL407337), which was consistent with the phylogenetic tree of 16S rRNA gene sequences (Figure 4B). The ANI values between *Bartonella* spp. (DTF8 and DTF9) and *B. rochalimae* (NZ KL407337) ranged from 91.79% to 94.52 %, indicating two uncultured new species within the genus *Bartonella*. The representative sequences of OPU 427 *Rickettsia* sp1 (1,897 reads in fleas) were shown to be clustered with *Rickettsia limoniae* (AF322442; Figure 5B), with an average identity of 97.23 ± 0.025 %, indicating that OPU 427 could be a new species affiliated to genus *Rickettsia*.

## Discussion

In the present study, we have described the microbiota community in ticks and fleas collected from *M. himalayana* in Qinghai Tibet Plateau, China. By using the metataxonomic analyses combined with shotgun metagenomics, we obtained species-level microbiota profiles of ticks and fleas, with 90.21% and 97.50% of the total reads annotated at the species level (known species and potentially new species), respectively. The metataxonomic method based on the full-length 16S rRNA gene amplicon sequencing had been applied in the investigation of the microbiota in vultures, Tibet antelopes, and human gut microbiota (Meng et al., 2017; Bai et al., 2018; Yang et al., 2020) in our previous studies. We identified a total of 1,250 and 689 OPUs (species-level taxon) in ticks and fleas, respectively. The major bacterial at phylum-level microbiota of ticks and fleas in our study included *Proteobacteria* and *Firmicutes*, followed by *Cyanobacteria* and *Actinobacteria*, which was consistent with the previous studies of fleas (Supplementary Figure 2; Hawlena et al., 2013), ticks (Andreotti et al., 2011; Thapa et al., 2019), and other vectors (Minard et al., 2013; Yun et al., 2014).

Notably, at the species level, we detected the known vector-borne pathogen *A. phagocytophilum* (OPU 421) and several novel species affiliated with vector-borne pathogenic genera, *Ehrlichia, Bartonella*, and *Rickettsia*, in the present study and performed deep analysis combined with metagenomic assembly and binning. The *A. phagocytophilum*, known as the causative agent of anaplasmosis, is responsible for approximately 6,000 cases of human granulocytic anaplasmosis per year in the USA (Bakken and Dumler, 2015). Previous studies have reported that the *A*. *phagocytophilum* has been divided into pathogenic *A. phagocytophilum* and the closely related nonpathogenic *A. phagocytophilum*-like (APL) species (Ben Said et al., 2017; Seo et al., 2018; Yan et al., 2022). From the phylogenetic analyses based on 16S rRNA gene sequences, the OPU 421 identified ticks in this study clustered with pathogenic *A. phagocytophilum* (Figure 3A), indicating that the ticks in the Qinghai-Tibet Plateau harbored pathogenic *A. phagocytophilum*. Recently, Duan et al. (2022) in their study detected the *A. phagocytophilum* for the first time in *M. himalayana* in Tibet, China, confirming that *M*. *himalayana* is a reservoir for *A. phagocytophilum*. Most importantly, they have found that plague may be exacerbated in the presence of *A. phagocytophilum* and humans that are at risk of infection by exposure to such marmots through ticks and fleas, potentially leading to a complicated disease.

In addition, using the phylogenetic analyses based on full-length 16S rRNA genes and core genes, we found that these novel species were most close to *E*. *muris, E*. *muris* subsp. *eauclairensis, B. rochalimae*, and *R. limoniae*, respectively. *E*. *muris* was originally isolated in 1983 and was identified as the cause of the patients in Minnesota and Wisconsin in 2009 (Pritt et al., 2011). In addition, the *E. muris* subsp. *eauclairensis* was recognized as the etiological agent of human ehrlichiosis in Minnesota and Wisconsin (Lynn et al., 2019). Although transmission to human appears to be limited to Minnesota and Wisconsin, this species could be a potential hazard to humans in the studied area. *Bartonella* were the most frequently identified bacteria in the fleas collected from rodents in southern Indiana, USA (Hawlena et al., 2013). *Bartonella rochalimae* can cause intra-erythrocytic infections in mammals and can cause disease manifestations that include bacteremia, splenomegaly, fever, and myalgia in humans. This pathogen has been identified in *Xenopsylla cheopis* fleas collected from *Rattus norvegicus* rats in Los Angeles, California (Billeter et al., 2011). *Rickettsia* can cause a group of diseases such as spotted fever group rickettsioses. However, with more *Rickettsia* species identified, plenty of *Rickettsia* were classified into endosymbionts rather a pathogen. *Rickettsia limoniae* has been reported from the microbiome of non-agricultural insects including the cranefly *Limonia chorea* (Perlman et al., 2006). However, its pathogenicity is unclear to date. In conclusion, these undescribed novel species, whether they can lead to diseases, need further study and surveillance.

Another important observation from our study was the detection of the vertically transmitted *Wolbachia* bacteria among these ticks and fleas in the present study. *Wolbachia* are obligate endosymbionts of bacteria that have been identified in various arthropods, including ticks, fleas, and mosquitos (Plantard et al., 2012; Duan et al., 2020; Flatau et al., 2021). Previous studies have reported that *Wolbachia* are essential for the survival and reproduction of the host and are involved in molting, embryogenesis, growth, and survival of the host. It can profoundly influence the ecology and evolution of its host through a wide range of symbiotic interactions. Currently, *Wolbachia* are the staple means of controlling vector-borne diseases due to their unique ability to spread through insect populations and to affect vector competence through pathogen protection. However, our understanding of their biology is still in its infancy and needs further study (Landmann, 2019; Manoj et al., 2021).

There are some shortcomings in this study. Although we obtained several metagenomic assembled genomes from ticks and fleas, we did not isolate potentially pathogenic species successfully. By controlling the density of marmots, the plague was rarely reported in the Qinghai-Tibet Platea in recent years. Thus, vector sample collection becomes more difficult, because it is only possible to collect ticks and fleas after a live marmot has been captured. This leads to another limitation in this study, which is a small sample size of ticks and fleas that we collected.

## Conclusion

By using the metataxonomic analysis based on full-length 16S rRNA gene amplicon sequencing, we comprehensively characterized the microbiota community in ticks and fleas collected from *M. himalayana* found in the Qinghai-Tibet Plateau at the species level for the first time. Notably, we identified the pathogenic *A. phagocytophilum* and potentially new vector-borne pathogens affiliated with genera *Ehrlichia, Bartonella*, and *Rickettsia*, and we performed deep analysis combined with metagenomic assembly and binning. The findings of the study have advanced our understanding of the potential pathogen groups of vectors in marmots in the Qinghai-Tibet Plateau, and we hope to provide etiological information for vector infectious disease prevention and control in the future.

## Data availability statement

The datasets presented in this study can be found in online repositories. The names of the repository/repositories and accession number(s) can be found at: https://www.ncbi.nlm.nih.gov/, PRJNA898891.

## Ethics statement

The animal study was reviewed and approved by Ethical Committee of National Institute for Communicable Disease Control and Prevention, Chinese Center for Disease Control and Prevention, China (No. ICDC-2018004).

## Author contributions

Conceptualization: LD and JY. Methodology: LD, JP, and JY. Data curation: JP and JY. Formal analysis: LD, JP, and CY. Visualization: LD, YL, CY, and JG. Writing—original draft: LD. Writing—review and editing: JY, JP, YL, WZ, YH, MK, LZ, and FW. All authors contributed to the article and approved the final version of the manuscript.
