# Supplementary material for: Species-level microbiota of ticks and fleas from Marmota himalayana in the Qinghai-Tibet Plateau
Source: Front Microbiol. 2023 Jun 21;14:1188155. doi: 10.3389/fmicb.2023.1188155 (PMC10320725; doi:10.3389/fmicb.2023.1188155)
Supplement: Supplementary file 1 [file Data_Sheet_1.zip › Contents of supplementary material.DOCX]

***Supplementary Material***

**Supplementary Figures**

**FIGURE S1.** **The relative abundance of OPUs in ticks and fleas.** The OPUs were classified into known species, putative new species, higher taxa, and uncultured bacterial.

**FIGURE S2. The stacked bar plot of relative abundance of top 10 phyla (A) and top 20 genera (B)**

**Supplementary Tables**

**Table S1. Information of samples enrolled the study**

**Table S2. Quality control yields of full-length 16S rRNA gene sequencing by PacBio**

**Table S3. The annotated information table of OPU**

**Table S4. Quality control data of metagenomic sequencing and assembly**

**Table S5. The information of metagenomic assembled genomes**
