# Supplementary figures and images for: Species-level microbiota of ticks and fleas from Marmota himalayana in the Qinghai-Tibet Plateau
Source: Front Microbiol. 2023 Jun 21;14:1188155. doi: 10.3389/fmicb.2023.1188155 (PMC10320725; doi:10.3389/fmicb.2023.1188155)

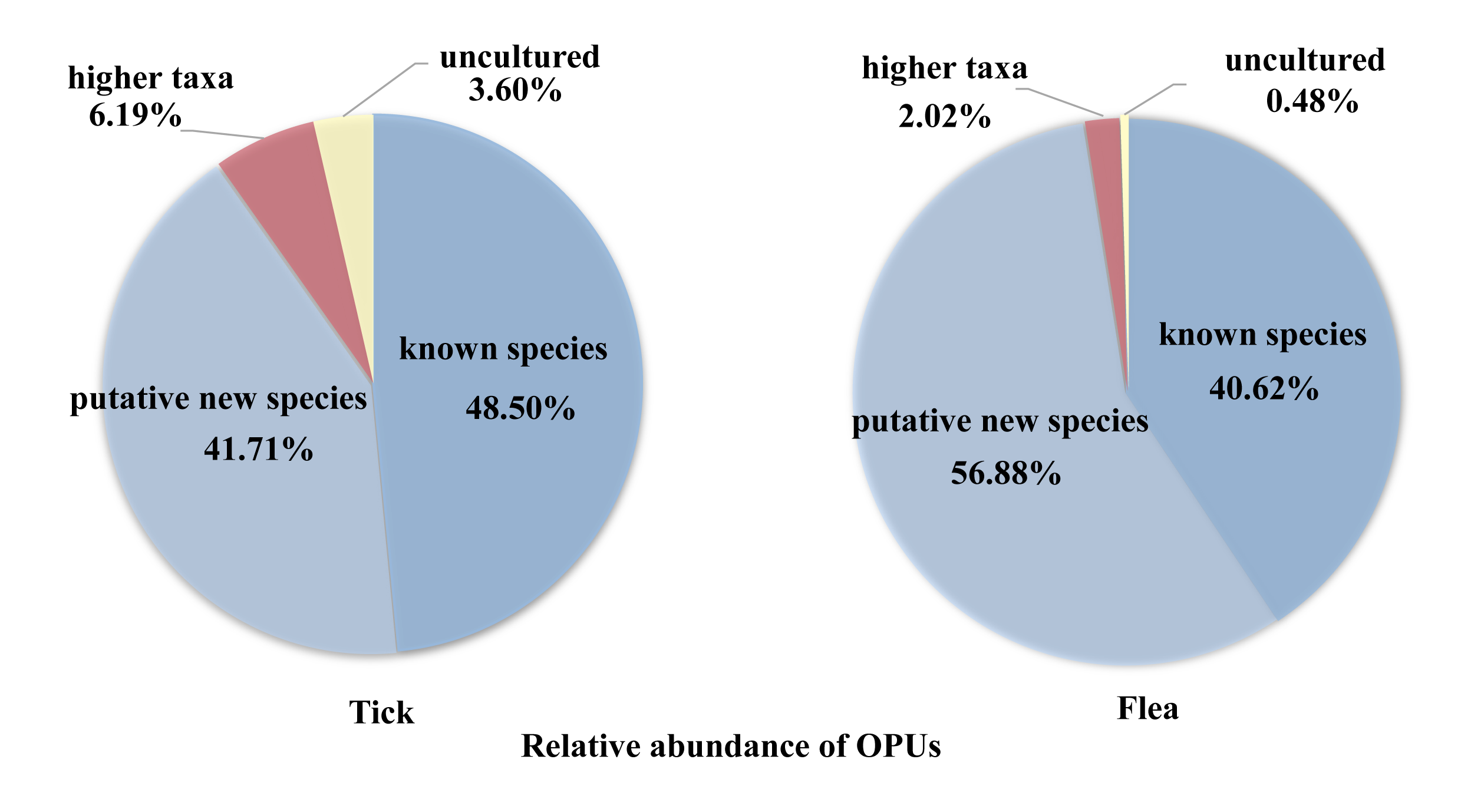

Supplement: Supplementary file 1 [file Data_Sheet_1.zip › Image S1.TIF]

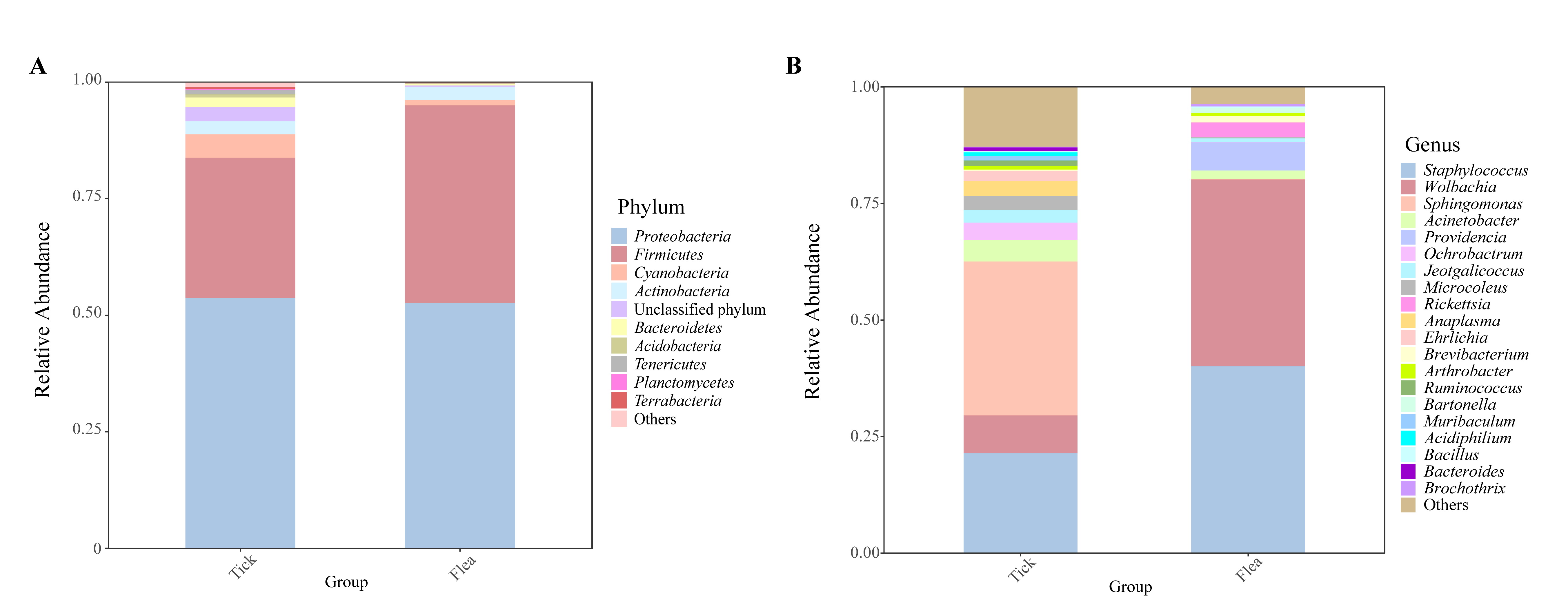

Supplement: Supplementary file 1 [file Data_Sheet_1.zip › Image S2.TIF]
